# Everest 60 years on: what next?

**DOI:** 10.1186/2046-7648-2-20

**Published:** 2013-06-06

**Authors:** Michael PW Grocott, Denny ZH Levett

**Affiliations:** 1 Integrative Physiology and Critical Illness Group, Clinical and Experimental Sciences, Sir Henry Wellcome Laboratories, Faculty of Medicine University of Southampton, Mailpoint 810, University Hospital Southampton NHS Foundation Trust, Tremona Road, Southampton SO16 6YD, UK; 2 Anaesthesia and Critical Care Research Unit, Mailpoint 27, D Level, Centre Block, University Hospital Southampton NHS Foundation Trust, Tremona Road, Southampton SO16 6YD, UK; 3 NIHR Southampton Respiratory Biomedical Research Unit, Southampton Centre for Biomedical Research, MP218, Southampton General Hospital, Tremona Road, Southampton, SO16 6YD, UK; 4 UCL Centre for Altitude, Space and Extreme Environment Medicine, Portex Unit, UCL Institute of Child Health, 30 Guilford Street, London, WC1N 1EH, UK; 5 NIHR University College London Hospitals Biomedical Research Centre, Tottenham Court Road, London, W1T 7DN, UK

**Keywords:** Altitude, Hypoxia, Everest, Sherpa, Critical care

##  

On 29 May 1953, Sherpa Tenzing Norgay and Edmund Hilary stood on the 8,848 m (29,029 ft) summit of Mount Everest, finally demonstrating that humans could overcome the physical and mental challenges required to conquer the world’s highest peak. The 60th anniversary of this event is sadly the first with no member of the original expedition alive, since the death of George Lowe on 20 March 2013 at the age of 89 [1].

The successful 1953 expedition followed seven British expeditions to the north side of Everest during the 1920s and 30s. Although unsuccessful, these early expeditions achieved impressive altitudes. On several occasions, climbers exceeded 8,000 m (26,246 ft) both with supplemental oxygen (1922, 8,320 m/27,300 ft) and without (1924, 8,570 m/28120 ft; 1938, 8,230 m/27,000 ft).

Following the Second World War and the subsequent assertion of Chinese control over Tibet in 1950, access to the north side of the mountain was limited and reconnaissance of the south side commenced in 1950 and 1951. In 1952, Tenzing Norgay Sherpa, climbing with Raymond Lambert on two Swiss expeditions, came tantalizingly close to the summit using supplemental oxygen (reaching 8,600 m/28,215 ft). The following year, Tenzing returned with John Hunt’s British expedition and, following the initial setback of Evans and Bourdillon’s failed attempt using a closed circuit oxygen delivery system, Tenzing and Hilary summited using an open circuit.

The successful 1953 expedition benefited from many lessons learned by the pre-war British, and post-war Swiss expeditions, including the benefits of supplemental oxygen, effective acclimatization strategies, equipment selection and detailed route knowledge. High altitude physiology research in the field was also important to the expedition’s success. In particular, knowledge gained from Griffiths Pugh’s studies on Cho Oyu in 1952, focusing on the design of oxygen delivery systems and the importance of adequate fluid intake at extreme altitude, may have been a critical success factor in 1953 [2].

Whilst Hilary and Tensings’ ascent confirmed that humans could overcome the physical climbing challenges of Everest, whether it would be possible without the assistance of supplemental oxygen remained a source of debate. Just over 35 years ago, on 8 May 1978, Reinhold Messner and Peter Habeler emphatically ended this debate. Intriguingly, this exceptional mountaineering performance (Habeler reportedly descended from the summit to the South Col in 45 min) contrasted with the climbers’ strikingly normal physiology [3].

Two years later, John West’s American Medical Research Expedition to Everest provided us with field data illustrating the profound physiological changes at such extreme altitude. They measured oxygen consumption at 6,300 m (20,669 ft) whilst breathing air and hypoxic mixtures equivalent to the summit of Mt. Everest [4] and expired gas samples from Chris Pizzo at the summit of Everest [5]. Twenty-six years after that, the Caudwell Xtreme Everest expedition team measured oxygen consumption at 7,950 m (26,082 ft) [6] on the South Col of Everest and obtained arterial blood gases close to the summit of Everest on the ‘balcony’ of the south-east ridge at 8,400 m (27,559 ft) [7]. Whilst these data are fascinating, they do not explain why some, such as Messner and Habeler, adapt so well to such altitudes, whilst others struggle.

So what does the future hold for Everest? What are the current challenges for integrative physiologists and physicians? We suggest some areas that may merit further exploration.

First, the de-coupling of systemic oxygen delivery from oxygen consumption during exercise that occurs after acclimatization merits better explanation [8]. This may represent auto-regulatory (microcirculatory) adaptation or cellular energetic changes (mitochondrial adaptation). Candidate causative mechanisms include the role of nitrogen oxides, including nitric oxide as a potential regulator of regional blood flow, microcirculatory function and mitochondrial function. Explaining the wide inter-individual variability in performance that occurs as a consequence of this phenomenon is likely to bring useful insights into hypoxic adaptation in other circumstances, including clinical diseases where hypoxia is an element (e.g. critical illness) [9].

Second, does repeated exposure to altitude alter subsequent adaptation? How important is early-life hypoxic exposure? Are epigenetic mechanisms in play? [10]

Third, the apparently striking differences between the performance of Sherpas (and other native highland races) and lowlanders deserves further evaluation. Comparisons between lowland clients and their Sherpa climbing companions are confounded by many factors. In particular, it is unclear what observations would come from the direct comparison of elite climbers from both groups.

Fourth, how can we improve our ability to predict altitude illness and performance at altitude? At present, the only reliable (but limited) predictor is how an individual responded during previous episodes of altitude exposure [11].

Fifth, what is the pattern of de-acclimatization? How long does acclimatization last? Is there harm associated with re-immersion in the relative hyperoxia of the low-altitude atmosphere (for example through generation of reactive oxygen species) [12]?

Sixth, the underlying assumption that pressure is not an important factor in altitude illness is not substantiated. It is generally accepted that the pathogenesis of altitude illness relates solely to hypoxic exposure, rather than having a significant contribution from occult ‘bubble disease’ or other pressure-related phenomena [13].

Seventh, and finally, the use of drugs in high-altitude mountaineering merits further consideration, from the ‘apparently’ harmless recommendation of routine prophylactic acetazolamide by some guides [14], through the breathing of supplemental oxygen high on Everest (which clearly improves safety [15]) to the more obviously unpalatable use of drugs for performance enhancement. This includes the widely acknowledged, but poorly documented, use of dexamethasone, amphetamines, nifedipine and other drugs in the absence of clinical disease. Should such drug use be encouraged in view of the safety considerations (and the lack of a safety net if altitude illness does develop)? Do such considerations apply equally to elite as well as recreational climbers and to Sherpas?

Sixty years on from the first ascent of Everest, we have an opportunity to reflect on the extraordinary history of physiological research and climbing on the mountain. On some occasions, physiologists have helped the climbers; whilst on others, we have been left to marvel at their (almost) inexplicable performance. Looking forward, we have suggested some areas that merit further exploration by future generations of high altitude physiologists and physicians.

## Competing interests

MPWG was the chief investigator of the Caudwell Xtreme Everest 2007 study and is the chair of the Caudwell Xtreme Everest Hypoxia Research Consortium. DZHL was the deputy research leader on the Caudwell Xtreme Everest 2007 study and serves on the Caudwell Xtreme Everest Hypoxia Research Consortium Executive Group. The authors declare that they have no other competing interests in relation to this article.
